# Development and validation of a predictive model for diabetic kidney disease risk in patients with T2DM: a hospital data platform study

**DOI:** 10.3389/fendo.2026.1868515

**Published:** 2026-06-30

**Authors:** ZhanLin Zhang, GuoXia Ma, MeiFang Ma, TianRong Guo, Yu Zhao, XiuSheng Cheng, Zhonglin Yan, ZhengMing Yin, Gangyi Wang, YongDong An

**Affiliations:** 1 Department of Public Health Management, The People’s Hospital of Linxia Hui Autonomous Prefecture, Linxia, Gansu, China; 2 Department of Gynecology, The People’s Hospital of Linxia Hui Autonomous Prefecture, Linxia, Gansu, China; 3 Department of Endocrinology, The People’s Hospital of Linxia Hui Autonomous Prefecture, Linxia, Gansu, China

**Keywords:** diabetic kidney disease, Internal validation, LASSO regression, logistic regression, nomogram, prediction model, temporal internal validation, type 2 diabetes mellitus

## Abstract

**Objective:**

This study used clinical data from patients with type 2 diabetes mellitus (T2DM) and applied least absolute shrinkage and selection operator (LASSO) regression to identify risk factors for diabetic kidney disease (DKD). We then constructed a nomogram prediction model to support early clinical screening of high-risk populations.

**Methods:**

Clinical data from patients with T2DM were collected from January 2020 to December 2025. Data from January 1, 2020, to December 31, 2023, were used as the training set for LASSO-based variable selection, model development, and internal validation. Data from January 1, 2024, to December 31, 2025, were used as a temporal internal validation set. Variables selected by LASSO regression were entered into a multivariable logistic regression model, and a nomogram was constructed from the regression results. Discrimination was assessed using the area under the receiver operating characteristic curve (AUC). Calibration was evaluated using calibration curves, and clinical net benefit was assessed using decision curve analysis (DCA). Internal validation was performed with 1,000 bootstrap replicates.

**Results:**

The retrospective study included 23,152 patients with T2DM, of whom 5,019 (21.68%) had DKD and 18,133 (78.32%) did not. LASSO regression identified nine candidate predictors: hypertension, diabetes duration, HbA1c, estimated glomerular filtration rate, urine protein, serum creatinine, uric acid, total cholesterol, and homocysteine. The nomogram showed moderate discrimination, with an AUC of 0.773 (95% CI: 0.764-0.782). In the temporal internal validation set, the AUC was 0.758 (95% CI: 0.743-0.774), indicating similar performance over time within the same hospital system. The calibration curves for both validation procedures were close to the diagonal, indicating agreement between predicted and observed probabilities.

**Conclusion:**

Based on real-world clinical data, this study used LASSO regression to identify risk factors associated with DKD in patients with T2DM. We developed a nomogram that integrates multidimensional predictors to estimate individualized DKD risk. Internal validation and temporal internal validation showed acceptable discrimination, calibration, and clinical net benefit. The model may serve as an auxiliary decision-support tool for early screening of patients at high risk of DKD in clinical practice. Because the AUC values indicate moderate rather than strong discrimination, multicenter external validation is still needed before broad implementation. The model is intuitive, practical, and cost-effective, and it may help clinicians identify high-risk patients who warrant closer monitoring or intensive intervention.

## 1 Introduction

Diabetes mellitus remains one of the most serious global public health challenges of the 21st century, and its prevalence continues to rise (1). According to the International Diabetes Federation, 589 million people aged 20–79 years had diabetes mellitus worldwide in 2024 (2). The burden of diabetes in China is particularly severe: prevalence increased rapidly from less than 1% in 1980 to 12.4% in 2018, and Chinese adults with diabetes account for approximately one-quarter of the global total (3, 4). Type 2 diabetes mellitus (T2DM) accounts for approximately 90%-95% of all diabetes cases (1). T2DM is not only a metabolic disorder characterized by hyperglycemia but also a chronic, progressive disease that can damage multiple organ systems, reduce quality of life, and impose a substantial economic burden (5, 6).

Diabetic kidney disease (DKD) is one of the most common and severe microvascular complications of T2DM and the leading cause of end-stage renal disease (ESRD), imposing a heavy burden on families and healthcare systems (7, 8). Studies indicate that approximately 20%-40% of patients with T2DM develop DKD (9). Once DKD progresses to ESRD, quality of life declines sharply and mortality increases substantially. Therefore, early identification of patients with T2DM who are at high risk of DKD, followed by timely and effective intervention, is essential for delaying or possibly halting progressive deterioration of renal function and improving patient outcomes (10).

Current DKD screening primarily relies on laboratory markers such as urinary albumin excretion, the urinary albumin-to-creatinine ratio (UACR), and estimated glomerular filtration rate (eGFR) (11). However, these markers may have limited sensitivity and specificity in the early stages of disease. For example, transient proteinuria may be induced by hypertension or strenuous exercise, making early warning difficult (12).

Although renal biopsy is the gold standard for confirming DKD, it is invasive and carries risks such as bleeding and infection. In addition, because renal biopsy requires specialized technical expertise and equipment, it is difficult to implement widely in the general diabetic population, particularly in primary care settings (10, 13). These challenges underscore the urgency and clinical value of developing a noninvasive, accurate, and readily deployable risk prediction tool for DKD.

With the development of digital health infrastructure and big data technology, clinical data mining based on hospital electronic medical record systems and laboratory information systems has provided a broad platform for disease prediction models (14). Real-world data allow researchers to systematically examine factors associated with disease. At the same time, advances in machine learning have made it possible to construct increasingly accurate prediction models.

Machine learning algorithms can handle high-dimensional and nonlinear data and therefore provide powerful tools for DKD prediction (15). However, the “black box” nature of many algorithms limits their clinical adoption and trustworthiness (16). In contrast, logistic regression combined with nomogram visualization can quantify the contribution of each predictor and provide individualized risk estimates. This approach balances accuracy, interpretability, and clinical utility and is a practical, widely accepted strategy for developing clinical decision-support tools (17).

Although significant progress has been made in research on DKD prediction models, important limitations remain. Firstly, most models were developed in specific populations, and the robustness and generalizability of their predictive performance require further validation (18, 19). Secondly, many models lack post-development evaluation of clinical utility, including decision curve analysis (DCA) to assess net benefit. This evaluation is critical for determining whether a model can benefit patients (17). Thirdly, limited applicability in primary care settings hinders widespread adoption of these models (19).

To address these limitations, this study has four distinctive features. First, the model was developed using real-world hospital data derived directly from routine clinical practice rather than from strictly screened research cohorts, thereby better reflecting actual diagnostic and treatment settings. Second, the model was designed for a Chinese T2DM population. All included predictors - hypertension, diabetes duration, HbA1c, eGFR, urine protein, serum creatinine, uric acid, total cholesterol, and homocysteine - are routine indicators that are widely available without specialized equipment, supporting use in primary care settings. Third, LASSO regression was used for variable selection to reduce overfitting, and a visual nomogram was constructed to facilitate individualized risk assessment. Fourth, beyond reporting discriminative performance using the area under the receiver operating characteristic curve (AUC), as most previous studies have done, this study evaluated calibration and clinical net benefit, providing more rigorous evidence for potential clinical translation.

This study aimed to use real-world clinical data from patients with T2DM obtained from the hospital information system (HIS) and laboratory information system (LIS) and to apply LASSO regression to identify factors associated with DKD. We sought to determine the optimal combination of routine indicators and evaluate their predictive performance. Based on readily available clinical variables, we aimed to develop and validate a DKD risk assessment model for patients with T2DM. The findings are expected to provide clinicians with a noninvasive, convenient, and accurate tool for early identification of patients at high risk of DKD. Such a tool may support timely intensive interventions, delay kidney disease progression, improve long-term prognosis, and reduce the incidence of ESRD and the associated socioeconomic burden.

## 2 Methods

### 2.1 Study design and data sources

This retrospective real-world data study followed the TRIPOD (Transparent Reporting of a Multivariable Prediction Model for Individual Prognosis or Diagnosis) guidelines. It aimed to construct and validate a risk prediction model for DKD in patients with T2DM.

The study data were obtained from the Clinical Research Data Platform of Linxia Hui Autonomous Prefecture People’s Hospital in Gansu Province. This platform integrates data from the HIS and LIS and covers clinical and diagnostic information for patients with T2DM treated across all clinical departments of the hospital. The platform enabled systematic extraction of patient visit data and provided a reliable data foundation for model development. The study protocol was reviewed and approved by the Ethics Committee of Linxia Hui Autonomous Prefecture People’s Hospital (Ethics Approval No. LZYY-LLSP-2025-26). All data access, processing, and analysis were conducted within the hospital information security framework and in accordance with patient privacy and data security protocols.

### 2.2 Study population

#### 2.2.1 Inclusion criteria

(1) Patients with T2DM treated at our hospital between January 1, 2020, and December 31, 2025; (2) age ≥18 years.

#### 2.2.2 Exclusion criteria

(1) Patients with type 1 diabetes, special types of diabetes, or gestational diabetes; (2) patients with severe functional failure of vital organs (e.g., heart, liver, or brain), malignant tumors, hematological disorders, or autoimmune diseases.

#### 2.2.3 Study population grouping

The study population was divided into a training set and a temporal internal validation set according to the visit time window. This approach ensured that the two datasets were independent and nonoverlapping, thereby meeting the statistical requirements for model development and temporal internal validation. The training set consisted of patients with T2DM treated at our hospital from January 1, 2020, to December 31, 2023; it was used for LASSO-based variable selection, prediction model construction, and internal validation. The validation set consisted of patients with T2DM treated at our hospital from January 1, 2024, to December 31, 2025; it was used for temporal internal validation of the model.

### 2.3 Variable definitions and data collection

#### 2.3.1 Outcome variable

DKD was ascertained from medical records and defined as follows: (1) persistent albuminuria (UACR ≥30 mg/g on at least two occasions over ≥3 months) and/or (2) persistently reduced eGFR (<60 mL/min/1.73 m^2^ for ≥3 months), with (3) a clinical diagnosis of diabetic nephropathy documented by the attending physician after exclusion of other causes of kidney disease (20).

#### 2.3.2 Study variables

Demographic characteristics: age, sex, nationality, smoking history, and drinking history.

Clinical characteristics: height, weight, body mass index (BMI), diabetes duration (DMtime), and history of hypertension.

Laboratory indicators: glycated hemoglobin (HbA1c), total cholesterol (TC), triglycerides (TG), high-density lipoprotein cholesterol (HDL), low-density lipoprotein cholesterol (LDL), serum creatinine (Cr), uric acid (UA), homocysteine (Hcy), alanine aminotransferase (ALT), aspartate aminotransferase (AST), urine protein (Upn), D-dimer (DDimer), total bilirubin (TBIL), indirect bilirubin (IBIL), and direct bilirubin (DBIL).

Calculated parameter: eGFR was calculated using the Chronic Kidney Disease Epidemiology Collaboration (CKD-EPI) equation (21).

### 2.4 Data preprocessing and quality control

To ensure data quality and model stability, raw extracted data were preprocessed under prespecified quality-control procedures. Outliers were detected using the boxplot rule (Tukey’s fences) combined with clinical judgment. Specifically, observations below Q1 − 1.5 × IQR or above Q3 + 1.5 × IQR were defined as potential outliers, where Q1 and Q3 represent the first and third quartiles and IQR denotes the interquartile range. All potential outliers were verified by reviewing original medical records to rule out data entry errors. Extreme values judged to be implausible or unrelated to pathophysiology were excluded, whereas plausible disease-related extreme values were retained to preserve clinical representativeness. Missing values were handled as follows: core variables (age, sex, diabetes duration, renal function indicators, and glucose and lipid metabolism indicators) with a missing rate >30% were excluded, and noncore variables with a missing rate >30% were also excluded. Variables with a missing rate ≤30% were imputed using multiple imputation.

Pearson correlation analysis and variance inflation factor (VIF) diagnostics were performed to evaluate multicollinearity among all indicators. A moderate negative correlation was observed between eGFR and serum creatinine (r = −0.613), with VIF values of 1.18 and 1.72 for serum creatinine and eGFR, respectively, indicating no substantial multicollinearity. Because eGFR and serum creatinine reflect related but not identical aspects of glomerular filtration function and creatinine metabolism, and because LASSO regression can reduce variable redundancy through the L1 regularization penalty, both indicators were retained in the final prediction model. Before variable selection by LASSO regression, all continuous predictors were standardized using Z-score normalization (i.e., centered to a mean of zero and scaled to unit variance). This step reduced the influence of differing units and measurement scales across variables. Standardization ensured that each predictor was treated more fairly during coefficient shrinkage in the L1 regularization algorithm, improved convergence of the L1 regularization algorithm, and increased the robustness of variable selection.

### 2.5 Model construction

#### 2.5.1 Variable selection method

LASSO regression was used for variable selection. By incorporating an L1 regularization term into the loss function, LASSO regression shrinks the coefficients of less informative variables to zero, thereby enabling variable selection (22). The optimal penalty coefficient λ was determined using 10-fold cross-validation, and predictors with nonzero coefficients were selected.

#### 2.5.2 Construction of the logistic regression prediction model

The variables selected by LASSO regression were used as predictors, and DKD occurrence was used as the outcome in multivariable logistic regression. The resulting model enabled individualized DKD risk assessment in patients with T2DM.

### 2.6 Model validation and evaluation

The prediction model was evaluated for discrimination, calibration, and clinical utility in both the training set (internal validation) and the temporal internal validation set. Discrimination was assessed using AUC. Calibration was evaluated by plotting calibration curves to compare predicted and observed probabilities. Clinical utility was assessed using DCA to estimate net benefit across a range of threshold probabilities. Internal validation was performed in the training set using 1,000 bootstrap resampling iterations to assess model stability. The temporal internal validation set was used to evaluate predictive performance in a later time period within the same hospital system.

### 2.7 Statistical analysis

All statistical analyses were performed using R version 4.4.2. Normally distributed continuous variables were expressed as the mean ± standard deviation and compared between groups using t-tests. Non-normally distributed continuous variables were expressed as the median (interquartile range) and compared using the Mann-Whitney U test. Categorical variables were expressed as frequencies and percentages and compared using the chi-square test.

## 3 Results

### 3.1 Characteristics of the study population

The study enrolled 23,152 patients with T2DM, comprising 5,019 (21.68%) in the DKD group and 18,133 (78.32%) in the non-DKD group. Patients in the DKD group were significantly older than those in the non-DKD group [65.00 (56.00, 73.00) years vs. 61.00 (53.00, 71.00) years, *P* < 0.001]. The proportion of males was also higher in the DKD group (62.52% vs. 58.28%, *P* < 0.001). Significant between-group differences were observed in ethnicity, marital status, drinking history, and smoking history (P < 0.05).

Regarding clinical characteristics, hypertension was more prevalent in the DKD group than in the non-DKD group (69.66% vs. 42.68%, *P* < 0.001), and diabetes duration was longer [8.00 (4.00, 12.00) years vs. 6.00 (3.20, 9.00) years, *P* < 0.001]. BMI did not differ significantly between the groups (P = 0.171). HbA1c levels were significantly higher in the DKD group than in the non-DKD group [9.20 (7.40, 11.20)% vs. 8.60 (7.10, 10.60)%, *P* < 0.001]. Homocysteine levels were also higher in the DKD group [15.00 (11.60, 19.55) μmol/L vs. 13.50 (10.70, 17.20) μmol/L, *P* < 0.001]. Significant differences were also observed in renal function indicators (e.g., eGFR, Cr, and UA), as well as in selected lipid profile and liver function indicators. Clinical characteristics of the two groups are compared in **Table 1**.

**Table 1:** Clinical characteristics of the study population [M (Q1, Q3) or n (%)] .

Variable	Total (23152)	NO DKD (18133)	DKD (5019)	Statistical value	*P* value
Age (years)	62.00 [54.00, 71.00]	61.00 [53.00, 71.00]	65.00 [56.00, 73.00]	-13.748	<0.001
Sex					
Male	13706 (59.20%)	10568 (58.28%)	3138 (62.52%)	29.11	<0.001
Female	9446 (40.80%)	7565 (41.72%)	1881 (37.48%)		
Nationality					
Han	9264 (40.01%)	7367 (40.63%)	1897 (37.80%)	35.522	<0.001
Hui	9080 (39.22%)	6942 (38.28%)	2138 (42.60%)		
Dongxiang	3804 (16.43%)	3033 (16.73%)	771 (15.36%)		
Tibetan	558 (2.41%)	426 (2.35%)	132 (2.63%)		
Other	446 (1.93%)	365 (2.01%)	81 (1.61%)		
Marital status					
Married	22073 (95.34%)	17325 (95.54%)	4748 (94.60%)	23.642	<0.001
Unmarried	184 (0.79%)	158 (0.87%)	26 (0.52%)		
Divorced/Widowed	895 (3.87%)	650 (3.58%)	245 (4.88%)		
Drinking history					
No	20868 (90.13%)	16278 (89.77%)	4590 (91.45%)	12.325	<0.001
Yes	2284 (9.87%)	1855 (10.23%)	429 (8.55%)		
Smoking history					
No	21387 (92.38%)	16651 (91.83%)	4736 (94.36%)	35.494	<0.001
Yes	1765 (7.62%)	1482 (8.17%)	283 (5.64%)		
Hypertension					
No	11917 (51.47%)	10394 (57.32%)	1523 (30.34%)	1144.163	<0.001
Yes	11235 (48.53%)	7739 (42.68%)	3496 (69.66%)		
DMtime (years)	6.00 [3.33, 10.00]	6.00 [3.20, 9.00]	8.00 [4.00, 12.00]	-22.528	<0.001
BMI (kg/m^2^)	24.02 [21.55, 26.12]	24.03 [21.60, 26.12]	23.94 [21.48, 26.12]	1.368	0.171
HbA1c (%)	8.70 [7.20, 10.70]	8.60 [7.10, 10.60]	9.20 [7.40, 11.20]	-12.008	<0.001
eGFR (mL/min/1.73 m^2^)	99.00 [85.00, 109.00]	100.00 [89.00, 110.00]	89.00 [55.00, 104.00]	34.589	<0.001
TG (mmol/L)	1.50 [1.08, 2.17]	1.49 [1.07, 2.14]	1.58 [1.13, 2.33]	-7.918	<0.001
LDL (mmol/L)	2.40 [1.87, 2.96]	2.40 [1.88, 2.94]	2.40 [1.83, 3.03]	-1.064	0.287
HDL (mmol/L)	1.29 [1.04, 1.59]	1.29 [1.04, 1.59]	1.29 [1.02, 1.61]	0.313	0.754
TC (mmol/L)	4.18 [3.42, 4.97]	4.16 [3.42, 4.93]	4.24 [3.44, 5.15]	-5.618	<0.001
Hcy (μmol/L)	13.80 [10.90, 17.70]	13.50 [10.70, 17.20]	15.00 [11.60, 19.55]	-17.822	<0.001
Upn	0.00 [0.00, 0.00]	0.00 [0.00, 0.00]	0.00 [0.00, 1.00]	-28.007	<0.001
Cr (μmol/L)	62.80 [51.60, 77.50]	61.00 [50.60, 73.00]	73.90 [57.20, 112.00]	-36.202	<0.001
AST (U/L)	19.00 [15.00, 27.00]	19.00 [15.00, 27.00]	19.00 [15.00, 27.00]	1.449	0.147
ALT (U/L)	23.00 [15.00, 38.00]	24.00 [16.00, 38.00]	22.00 [15.00, 35.00]	5.803	<0.001
UA (μmol/L)	281.80 [219.20, 355.70]	273.30 [213.80, 341.90]	318.80 [247.00, 410.25]	-26.077	<0.001
DDimer (μg/mL)	0.30 [0.14, 0.68]	0.28 [0.14, 0.63]	0.36 [0.17, 0.88]	-12.108	<0.001
TBIL (μmol/L)	13.70 [9.60, 19.50]	14.00 [10.00, 19.90]	12.10 [8.10, 17.70]	17.647	<0.001
IBil (μmol/L)	9.10 [6.10, 13.30]	9.50 [6.40, 13.70]	7.80 [4.90, 11.90]	19.773	<0.001
DBIL (μmol/L)	4.30 [3.00, 6.30]	4.40 [3.00, 6.40]	4.00 [2.70, 6.00]	8.565	<0.001

### 3.2 Feature selection using LASSO regression

**Figure 1:**
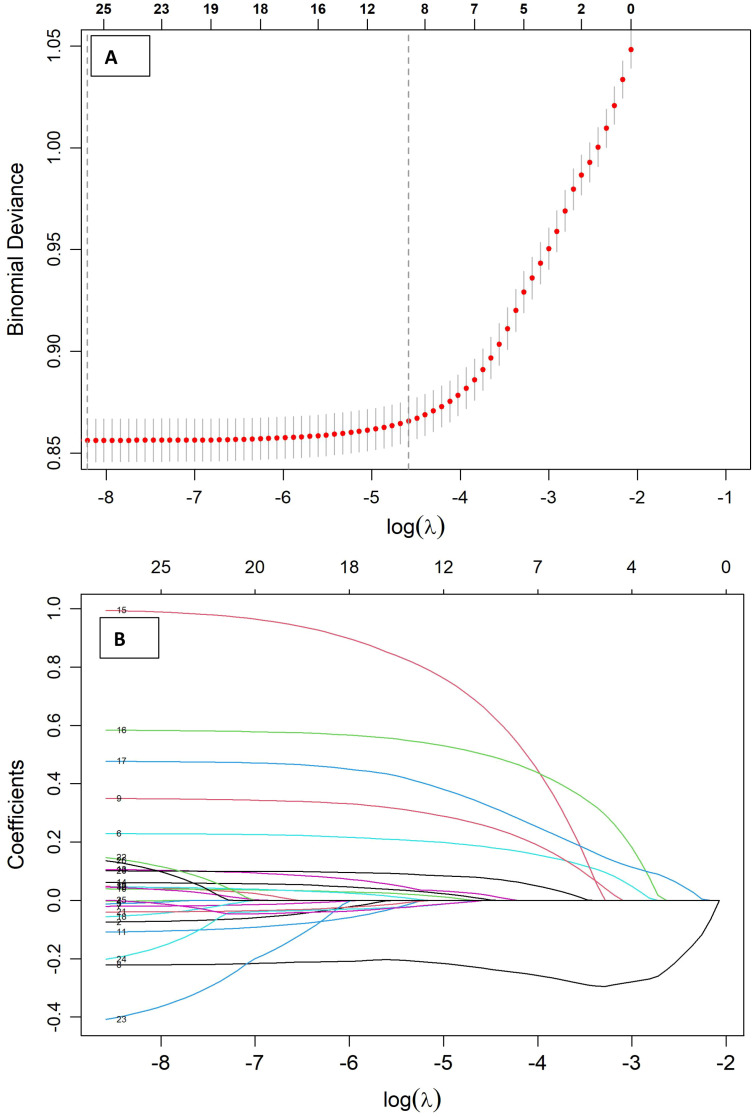
Feature selection based on LASSO regression. **(A)** Ten-fold cross-validation plot for LASSO regression. **(B)** Regularization path of LASSO regression coefficients.

In the training set, 25 candidate clinical and laboratory variables were considered. LASSO regression with 10-fold cross-validation was performed for feature selection. The optimal λ (lambda.1se) was defined as the λ value within one standard error of the minimum cross-validated error (**Figure 1**). Using this criterion, nine variables with nonzero coefficients were retained: hypertension, diabetes duration, HbA1c, eGFR, urine protein, serum creatinine, uric acid, total cholesterol, and homocysteine.

### 3.3 Development of the predictive model

**Figure 2:**
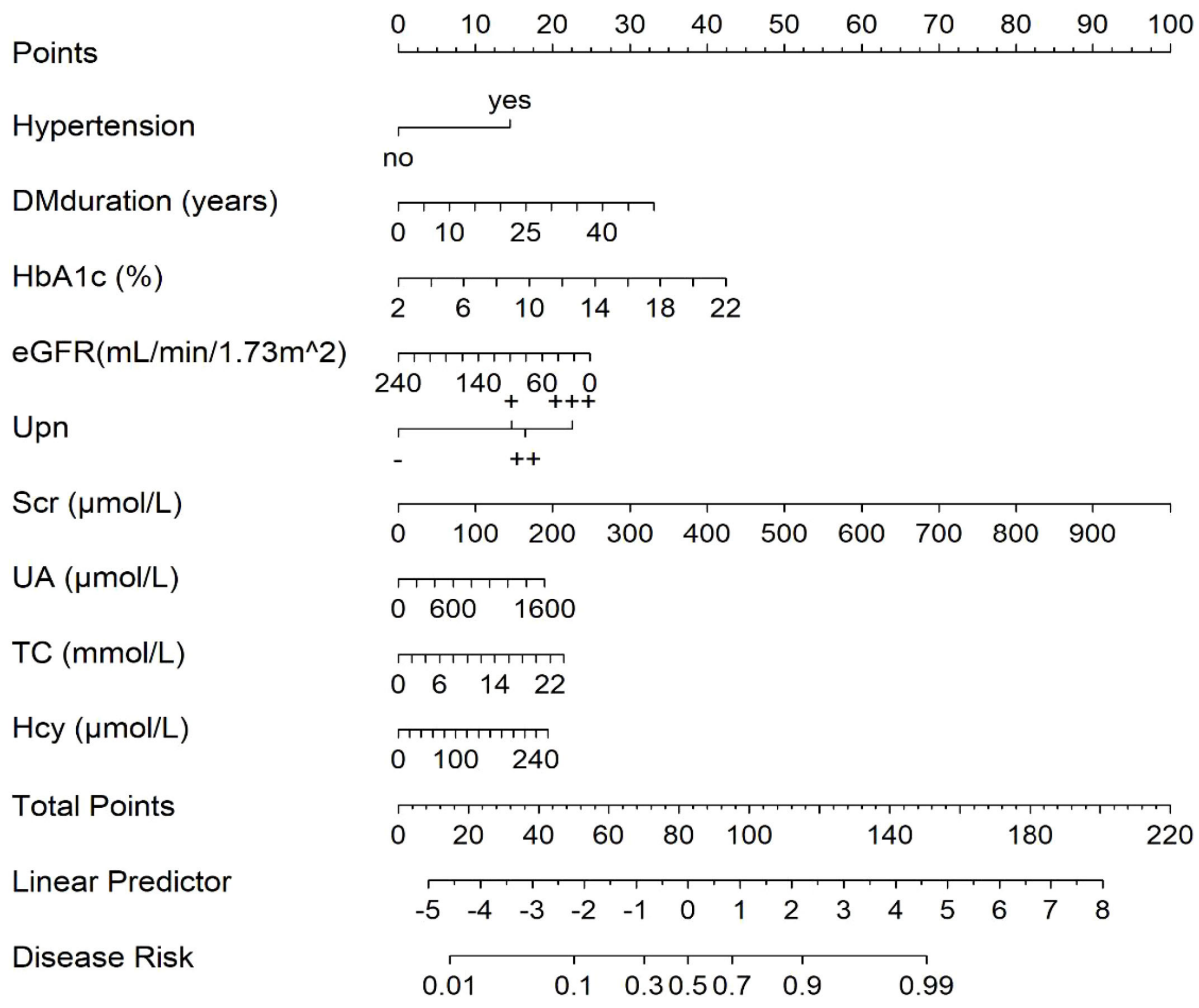
Nomogram for predicting DKD risk in patients with T2DM.

Multivariable logistic regression was performed using the nine predictors screened by LASSO regression as independent variables, with DKD occurrence set as the dependent variable. Based on the logistic regression results, we developed a nomogram to predict DKD risk in patients with T2DM. In the nomogram, each predictor is assigned a point value. Total points are summed, and the corresponding predicted probability of DKD is obtained by projecting the total score onto the risk axis (**Figure 2**).

### 3.4 Internal validation of the model

**Figure 3:**
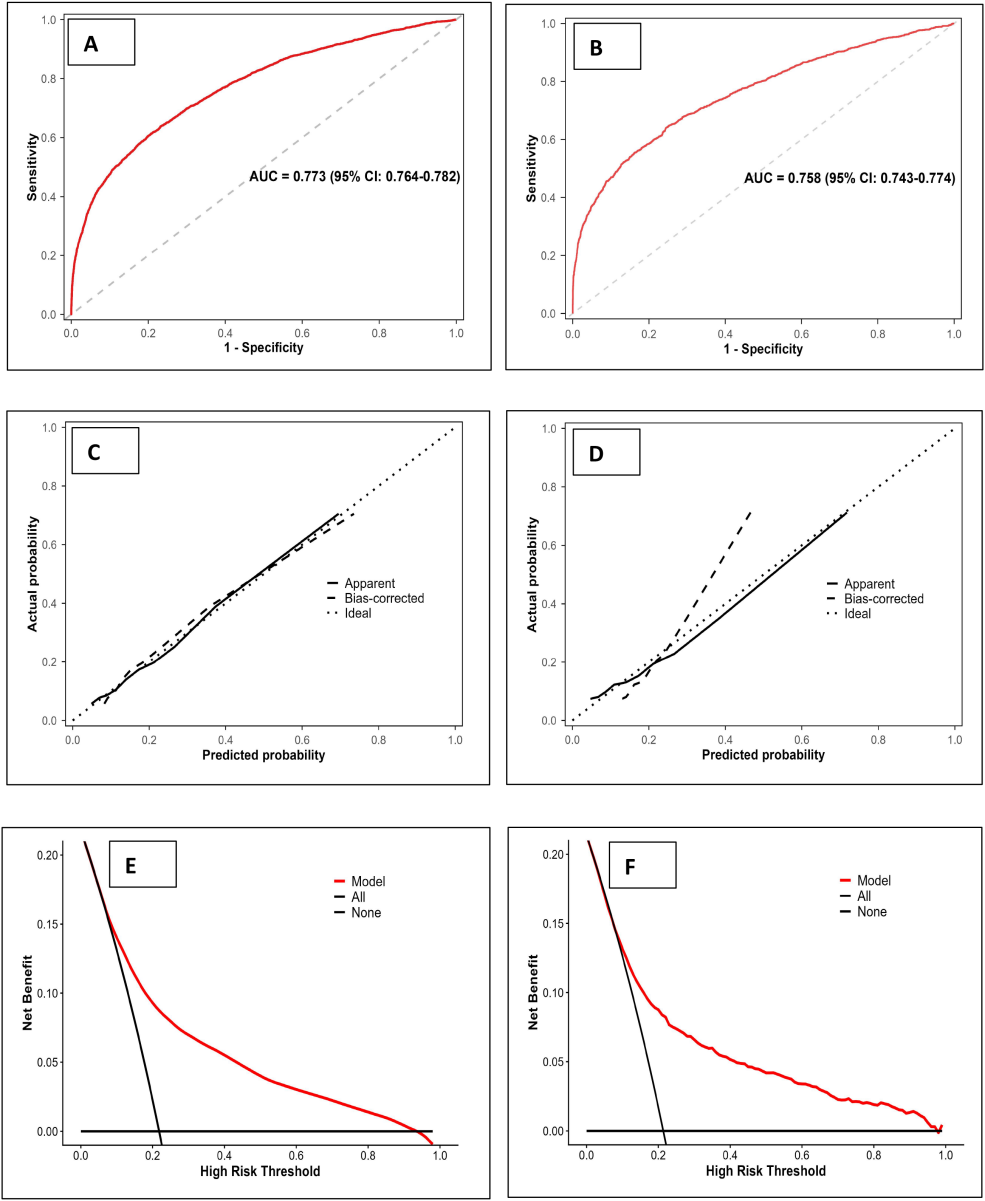
Performance evaluation of the DKD risk prediction model in patients with T2DM. **(A, C, E)** Internal validation results; **(B, D, F)** Temporal internal validation results.

The model was internally validated using 1,000 bootstrap resamples, yielding an AUC of 0.773 (95% CI: 0.764-0.782) (**Figure 3A**). This value indicates moderate discrimination. In the calibration curve, the x-axis represents model-predicted probability and the y-axis represents observed probability. A calibration curve closer to the diagonal indicates better agreement between predicted and observed probabilities. In this study, the calibration curve was close to the ideal curve (**Figure 3C**), indicating acceptable calibration. Decision curve analysis was used to assess clinical utility by comparing net benefit across treatment-decision thresholds. The horizontal axis represents the threshold probability, and the vertical axis represents net benefit. The thick black solid line indicates no intervention for any patient, the thin black solid line indicates intervention for all patients, and the red curve represents the net benefit of the prediction model across the threshold range. Across a relevant range of threshold probabilities, the model yielded greater net benefit than both the “All” and “None” strategies, indicating potential practical value (**Figure 3E**).

### 3.5 Temporal internal validation of the model

Temporal internal validation was performed to assess the reliability of the prediction model in a later set from the same hospital system. The model showed similar performance in this set, with an AUC of 0.758 (95% CI: 0.743-0.774) (**Figure 3B**), again indicating moderate discrimination. The calibration curve showed agreement between predicted and observed probabilities (**Figure 3D**), suggesting acceptable calibration in the temporal internal validation set. The decision curve analysis (**Figure 3F**) showed results similar to those observed in internal validation and indicated favorable net clinical benefit, supporting temporal robustness and potential clinical utility within the development setting.

## 4 Discussion

In this study, LASSO regression was used to identify risk factors for DKD in patients with T2DM, and multivariable logistic regression was used to develop a DKD risk prediction model. Nine predictors were identified: hypertension, diabetes duration, HbA1c, eGFR, urine protein, serum creatinine, uric acid, total cholesterol, and homocysteine. These variables are established or plausible risk factors for DKD in patients with T2DM (23). The model was evaluated using AUC, calibration curves, and DCA to assess discrimination, calibration, and clinical utility, respectively. Internal validation and temporal internal validation showed moderate discrimination, acceptable calibration, and favorable clinical net benefit. Overall, the model showed moderate but potentially useful predictive performance in both validation procedures.

Compared with previously reported prediction models for DKD, this study incorporated several practical optimizations. Many prior models were developed in highly selected research cohorts, relied primarily on AUC for performance evaluation, or included predictors that are not readily available in routine clinical practice. These factors limit generalizability and application in primary care settings. In contrast, our model was developed using real-world hospital data, incorporated widely accessible routine laboratory indicators, used LASSO regression for variable selection, and provided visual risk estimation through a nomogram. These features make the model adaptable to real-world screening scenarios and may help primary care clinicians perform individualized DKD risk assessment in patients with T2DM.

Variable selection in this study used LASSO regression, an effective method for identifying relevant variables in high-dimensional data. By applying regularization to all coefficients, LASSO regression shrinks the coefficients of relatively unimportant independent variables to zero and enhances model stability (22). This method has been widely applied in medical prediction models. Li also used LASSO regression in a large-scale study of patients with T2DM and identified 15 variables associated with DKD (24). Notably, our model overlapped with the variables identified by Li (24), including hypertension, serum creatinine, uric acid, HbA1c, and urine protein. However, our model is more concise, with only nine core variables, which may improve clinical utility and ease of implementation.

The distinctive feature of this study’s variable selection is the simultaneous inclusion of multiple indicators reflecting different dimensions of renal function (eGFR, urine protein, and serum creatinine). This multidimensional assessment may help capture different stages and mechanisms of renal dysfunction. This approach aligns with recent trends in proteomics research, such as the “eGFR + Five-Protein Panel” model developed by Xu, which combines eGFR with five protein biomarkers and shows strong performance in predicting diabetic chronic kidney disease (25). Although our model does not include emerging biomarkers, its comprehensive assessment strategy based on routine indicators follows a similar clinical logic.

Some recent studies have explored more complex multimodal models, such as the model developed by Shao that combined magnetic resonance imaging with serum and urine biomarkers and achieved high discriminative ability (26). However, the cost and operational complexity of such models may limit widespread implementation. Our model provides a more practical solution for routine clinical settings.

The prediction model in this study had an AUC of 0.773 in internal validation and 0.758 in temporal internal validation, indicating moderate discrimination. The DKD risk prediction model constructed by Yong included 10 indicators and had an AUC of 0.689 in the validation set (27). Although some predictors overlapped with those in our model, including HbA1c and serum creatinine, the performance of our model was comparable to or slightly better than that of several previously reported models. Some variables in this study, including HbA1c, serum uric acid, eGFR, urine protein, and serum creatinine, were also used in the models established by Belur Nagaraj (28) and Cheng (29). The present model did not include age or sex, consistent with the findings of Sun (30). Compared with the risk prediction model constructed by Deng (17) (AUC = 0.698), our model also showed higher discrimination. Nevertheless, its discriminative performance remained moderate, so further external validation is warranted prior to routine clinical application.

This prediction model suggests that patients with T2DM and longer diabetes duration may require earlier renal-protective measures to reduce DKD risk. Studies have shown that diabetes duration is closely associated with the onset and progression of DKD (31). As diabetes progresses, pancreatic islet cell number and function may decline. Poor long-term glycemic control may injure the renal vascular system and accelerate deterioration of renal function (32). Wang found that diabetes duration is an independent risk factor for the onset and progression of DKD (33). As disease duration increases, DKD risk also increases. Clinicians should therefore maintain heightened vigilance and perform early DKD screening in patients with long-standing T2DM.

This study found that hypertension was a risk factor for DKD in patients with T2DM, consistent with the findings of Zhou (34). Persistent elevation of blood pressure can activate the renin-angiotensin-aldosterone system and increase intraglomerular pressure. Sustained hyperperfusion may further contribute to renal arteriosclerosis, vascular wall thickening, and luminal narrowing. These changes reduce renal blood flow, worsen renal injury in patients with T2DM, and increase the risk of DKD (35). HbA1c is a key indicator of long-term glycemic control in patients with diabetes and plays an important role in DKD development (36). Previous studies have considered HbA1c a predictive marker for DKD (23). The model constructed in this study further suggests that HbA1c contributes to DKD risk prediction in patients with T2DM.

Meta-analyses indicate that higher serum uric acid levels in patients with T2DM are associated with increased DKD risk (9), and serum uric acid may serve as a predictor of DKD in these patients (20). Previous studies have shown that uric acid-lowering therapy can reduce urine protein and slow decline in renal function (37). This association may be related to the effects of serum uric acid, which can directly induce renal inflammation, fibrosis, glomerular hypertension, and microvascular endothelial damage (38), as well as to insulin resistance, oxidative stress, and fructose metabolism disorders in T2DM (39). This study also found a positive correlation between serum creatinine and DKD risk.

In our study, lower eGFR was closely associated with DKD development, consistent with previous research (40). This finding indicates that declining eGFR is an important predictor of DKD progression. Urine protein is a recognized biomarker of DKD progression (41). When renal function is severely impaired, increased glomerular permeability allows proteins to enter the urine, leading to elevated urine protein levels (42). The results of this study also indicate that urine protein is a risk factor for DKD in patients with T2DM. Urine protein played a significant predictive role in the model, supporting its clinical value for early DKD detection, consistent with previous findings (43).

Furthermore, the nomogram developed in this study provides clinicians with an intuitive and user-friendly risk assessment tool. Based on routine laboratory parameters, the model is cost-effective and accessible, making it suitable for primary care settings and resource-limited regions (44). Clinicians can use routine clinical data from patients with T2DM to identify those at high risk of DKD and implement targeted monitoring and intervention strategies.

This study has several limitations. Firstly, this was a single-center retrospective study, which did not account for potentially relevant factors such as living environment and dietary habits in patients with T2DM. This may have introduced information bias or selection bias. Secondly, we did not benchmark the logistic regression model against other machine learning algorithms (e.g., Random Forest or XGBoost). Logistic regression was selected because it is clinically interpretable and can be directly converted into a nomogram suitable for primary care practice. Finally, the predictive performance of this model was derived from a general T2DM population, which may limit its applicability in special populations with genetic or vasculopathic conditions, including Moyamoya disease. Moyamoya disease may be accompanied by renal artery stenosis and renovascular hypertension in some patients, and these conditions are established modifiers of DKD risk. In addition, systemic vasculopathy in Moyamoya disease may impair kidney function through nontraditional pathways, leading to potential overestimation or underestimation of DKD risk when the model is applied to patients with both T2DM and Moyamoya disease.

Future studies should conduct rigorous external validation using independent datasets, particularly by including rare subgroups such as patients with Moyamoya disease, to comprehensively assess the model’s predictive performance in these specific populations. In addition, multicenter, longitudinal cohort studies are needed to further improve the model’s predictive accuracy and generalizability. Furthermore, multiple machine learning algorithms (e.g., Random Forest and XGBoost) should be introduced to perform comparative model performance analyses.

## 5 Conclusion

In summary, this study established a DKD risk prediction model for patients with T2DM based on hypertension, diabetes duration, HbA1c, estimated glomerular filtration rate, urine protein, serum creatinine, uric acid, total cholesterol, and homocysteine. The model demonstrated modest but clinically useful predictive capability and utility. This model enables clinicians to identify high-risk patients at an early stage and deliver timely interventions, thereby improving patient outcomes.

## Data Availability

The original contributions presented in the study are included in the article/supplementary material. Further inquiries can be directed to the corresponding author/s.
